# Single-phase to three-phase DC-link boost converter with reduced controlled switch count

**DOI:** 10.1038/s41598-026-53542-z

**Published:** 2026-05-25

**Authors:** Heba Abdellatif Nagi, Awad E. El-Sabbe, Dina S. M. Osheba

**Affiliations:** 1 Department of Electrical Engineering, Faculty of Engineering, Menoufia University, Shebin El-Kom, Egypt; 2 Department of Electrical Engineering, Faculty of Engineering, Menoufia University, Shebin El-Kom, Egypt

**Keywords:** AC–DC–AC power converters, PWM, SPTTP conversion, Energy science and technology, Engineering

## Abstract

Three-phase motors are used in a variety of applications, such as industrial machinery, water pumping stations, and electric vehicles and transportation systems. They may be easily operated from a single-phase power source by using a suitable power converter to drive three phase motors from single-phase ac source. This paper proposes a single-phase to three-phase (SPTTP) converter with boosting capability of three-phase output voltages. The proposed configuration reduces the number of switches which is only six semiconductors and increasing the three-phase output voltages of single-phase ac source. Moreover, it improves the input and output currents profile of total harmonic distortion (THD) without using another switches in rectifier side. The fewer semiconductor switches and passive components leads to lower power losses and improved efficiency. The toggling control strategy employs a straightforward logic control circuit that is dependent on Sinusoidal Pulse Width Modulation (SPWM) technique. The modulation technique is simple approach rather than Space Vector Pulse Width Modulation (SVPWM) which used in the compared topologies. The architecture’s robustness and effectiveness are verified through the use of MATLAB/SIMULINK. The simulation analysis is verified by setting up and testing a prototype for this topology in the laboratory using a dSPACE-1104. Finally, the desired circuit is approved by the simulation and experimental results, which are examined by the good similarity under a variety of conditions.

**Supplementary Information:**

The online version contains supplementary material available at 10.1038/s41598-026-53542-z.

## Introduction

Single-phase to three-phase power converters are often used in distant and remote regions where distributing three-phase power supplies might be difficult. The simplest basic single-phase to three-phase converter is constructed from a three-leg (3L) inverter and a full bridge uncontrolled rectifier^1^. The standard single-phase configuration to three-phase converter employs six switches and diode bridge rectifier. The most straightforward method to convey power from a single-phase AC source to a three-phase AC demand is to employ a first-stage diode bridge to generate a DC link voltage with minimal ripples. The second stage which the three-phase inverter is fed by the DC link voltage to energize the load. The unregulated rectifier is linked to an inadequate grid current profile and a diminished power factor^2,3^. To improve the input power factor and operate at the grid side with unity power factor (UPF), the diode bridge is replaced with single-phase four-controlled switches. The primary disadvantage of this architecture is the higher converter cost, which results from the need for more gate controllers and power semiconductor devices to run the switches. The contemporary advancements in one-phase to three-phase converters are analyzed in^4^. This article illustrates two sorts of setups. A reduction in the number of components leads to a corresponding possible drop in the initial cost of the converter and a deterioration in the waveform’s quality. An increase in the number of components directly elevates the cost of the converters and enhances the quality of the waveform. The traditional architecture and topologies with a lower switch count have been analyzed in^5,6^ but with lower power density and a decline in the quality of the waveforms.

Single-stage single-phase AC to three-phase AC matrix converters were introduced in^7,8^ to enhance power density and longevity. These converters eliminate the dc-link capacitor. In order to eradicate the electrolytic capacitor^9^, illustrates a single-stage, single-phase AC to three-phase AC converter. The utilization of massive tapped coupled inductors at the connection has a detrimental impact on the input and output power factor of the converter. An increase in double-line frequency fluctuations in the link voltage is the result of the reduction or removal of the capacity of the DC link electrolytic capacitor. This results in elevated total harmonic distortion and undesirable harmonics at the input and output. As a result of this, a single-phase to three-phase power conversion system including parallel rectifier and a series inverter for each rectifier was suggested in the study^10^ and known with ten legs (10L). Such a converter ensures that the output voltage handled by the inverter circuit is reduced as well as the input current processed by the rectifier circuit^10^. Moreover, in^11,12^, a parallel complete bridge rectifier circuit is integrated to evaluate a SPTTP converter system with seven legs (7L) as a method of reducing the current management by rectifier switches. This arrangement improves harmonic distortion and efficiency on the rectifier side while reducing variations in the DC link electrolytic capacitor throughout the link voltage. The studies^13,14^ address a single-phase to three-phase AC-DC-AC system that contains two parallel rectifiers and two Space Vector Modulation (SVM)-based control schemes. These approaches specify the optimal choice for switching states in order to decrease the circulation current and regulate the grid current with minimum harmonic distortion. However, these are 10L and 7L design including twenty and fourteen power switches, respectively. The main drawback of these topologies is the increased cost of the converter, which is due to the increased number of power semiconductor devices and gate controllers required to operate the switches. In study^15^ describes an economical two legs (2L) single-phase to three phase converter which gives variable output voltage by employing four switches, four diodes, and a triac. It can produce and control the required DC link voltage. This increases unwanted harmonics at the input and output currents. The voltage gain of DC link capacitors in single-phase AC to three-phase AC converters has been the focus of certain studies^16,17^. These publications provide a comprehensive description of the conventional five-leg (5L) converter. The parallel five-leg (P5L) converter and the parallel four-leg (P4L) converter are two innovative topologies that have been introduced in these papers. The DC link voltage gain of the P5L and P4L topologies is preferable to that of the traditional 5L architecture. Since the topologies lack an augmenting element and have a greater number of components, the system is more complex, costly, and larger. However, the topologies of DC-DC converter in studies^18–22^ have also boosting factor of DC voltage but a higher count of components. Additionally, the DC link capacitor voltage gain increases the voltage stress on the inverter’s switches and increases the converter cost according to the following cost function (CF) equation:

$CF=\left({N}_{SW}+{N}_{driver}+{N}_{D}+{N}_{CAP}+\frac{\alpha {\left(TSV\right)}_{pu}}{Gain}\right),$ where the weight coefficient factor (α) can be close to one. TSV (total standing voltage) is the cross voltage on all switches which equals the DC voltage. TSV is calculated as:

$TSV=\frac{{\sum}_{i=1}^{n}{V}_{bswitch,i}+{\sum}_{j=1}^{m}{V}_{bdiode,j}}{{V}_{omax}} ,$ where V_bswitch, i_ and V_bdiode, j_ represents the maximum blocking voltage across each switch and diode respectively, and V_omax_ is the peak value of output voltage in the given topology^23^. A novel single-phase ac to three-phase ac converter that employs a tiny film capacitor in place of big electrolytic capacitors is shown in the publications^24,25^. The double-line frequency harmonic is absent from the input and output currents/voltages even when a small film capacitor is used. However, it has a lot of ripples in the capacitor voltage. The major shortcoming of all architectures is that the output terminal does not exhibit any voltage gain.

This paper offers the development of a SPTTP DC-link converter that is capable of enhancing the three-phase output voltages and employs a reduced number of controlled switches. The suggested converter attains a substantial output voltage gain while utilizing fewer components than comparable contemporary architectures disclosed in the literature. The suggested converter provides excellent efficiency and reduces converter power losses by using a small number of passive components and semiconductor switches. Moreover, it can be applied with straightforward PWM control technique. Furthermore, it enhances total harmonic distortion’s (THD) of the input and output current profile. This work is structured into nine sections subsequent to this introduction. Section “Proposed circuit” will give the model for the suggested converters. Section “System mathematical model” delineates the mathematical paradigm for topology. Section “Modulation technique” addresses pulse width modulation (PWM) techniques for the topology. Therefore, the loss calculations for the suggested arrangement are detailed in Sect. “Calculation of power losses and efficiency”. Section “Comparison study of single phase to three phase converter”, which presents the comparison study with previous different configurations. The simulation findings are shown in Sect. “Simulation results”. Section “Experimental results” analyzes the experimental outcomes. The conclusions are ultimately stated in Sect. “Conclusion”.

## Proposed circuit

**Fig. 1:**
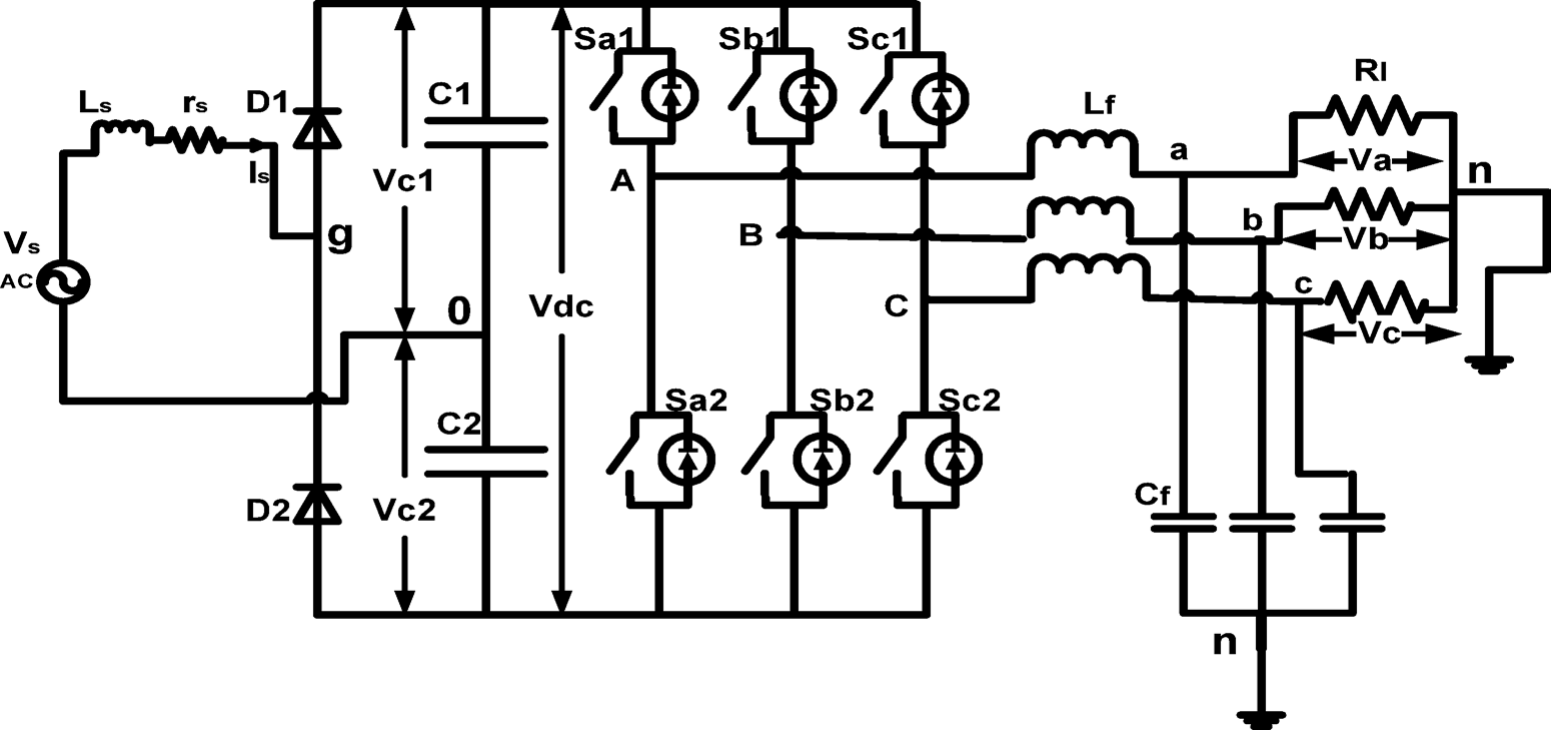
proposed circuit configuration.

The circuit layout of the suggested converter is depicted in Fig. 1. This suggested structure has three legs including six controlled switches, one leg containing two diodes, and one leg incorporating two capacitors as a DC link. The inductor-capacitor filters are used to improve the load voltage waveforms. The diodes D1 and D2 form the rectifier, two split capacitors create the dc-link, and the output inverter converts the dc-voltage to a balanced three-phase output with controllable voltage and frequency. Initially, the parallel diodes must charge the two capacitors (C1 and C2) to the power supply’s peak voltage at the positive and negative half cycles. The size of the two capacitors must be such that they appear as a nearly constant source of dc voltage with minimal ripple.

The primary switches Sa1, Sb1, and Sc1, along with their corresponding complementary bottom switches Sa2, Sb2, and Sc2, are the controlled legs. Each switch functions for 180° conduction modes in the regulated method. At any point, three switches are functional. The activation of switch Sa1 engages the DC voltage input’s positive terminal. Switch Sa2 is engaged, and the negative terminal of the input DC voltage is connected. Each cycle consists of eight operational modes, each functioning for 60°. Two of these states result in zero ac line voltage at the output, where the ac line currents freewheel via the upper or lower elements. The other states do not produce zero ac output line voltages; the inverter alternates between states to create a certain voltage waveform, so the resulting ac output line voltages are composed of discrete voltage values, namely − V_dc_, 0, and V_dc_. The sequence of gating pulses dictates the numbering of switches. Table 1 illustrates the transition pattern, with the ON state denoted as (1) and the OFF state as (0).

**Table1:** The switching states of the circuit.

State No	Switching state						V_AB_	V_BC_	V_CA_
	Sa1	Sa2	Sb1	Sb2	Sc1	Sc2			
1	1	0	0	1	0	1	V_dc_	0	− V_dc_
2	1	0	1	0	0	1	0	V_dc_	− V_dc_
3	0	1	1	0	0	1	− V_dc_	V_dc_	0
4	0	1	1	0	1	0	− V_dc_	0	V_dc_
5	0	1	0	1	1	0	0	− V_dc_	V_dc_
6	1	0	0	1	1	0	V_dc_	− V_dc_	0
7	1	0	1	0	1	0	0	0	0
8	0	1	0	1	0	1	0	0	0

## System mathematical model

The converter design depicted in Fig. 1 comprises a 3-phase inverter, a 3-phase output, a DC link, and a single-phase half-bridge rectifier.

**A. Rectifier model:**

The model that follows is derived from Fig. 1:

$$Vs = rs is + Ls dis/dt + Vgo$$

where r_s_ and L_s_ are considered source impedance but with small value thus can be ignored, V_go_ is the pole voltage of the rectifier between points (o) and (g) in Fig. 1.

1 $$Vs=Vgo$$

2 $$Vgo = qd Vc1 {-} \left( {1 - qd} \right) Vc2$$

3 $$Vc1 = Vc2$$

4 $$Vdc = Vc1 + Vc2=2Vc$$

Consequently, the two diodes operate as a bi-directional switch with dual poles. This switch is effectively represented as a toggling logical variable q_d_. By substituting for V_dc_ in Eq. (2), the differential equations for the circuit can be expressed as:

5 $$Vgo = (2qd -1) Vdc/2$$

**Fig. 2:**
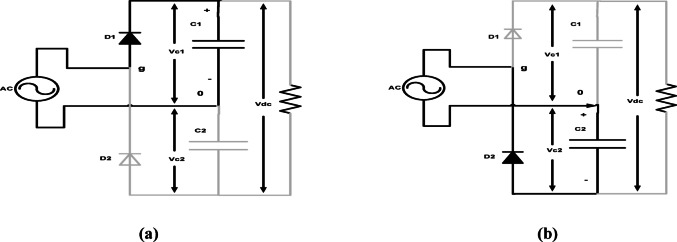
Operational mode for rectifier circuit: (**a**) positive input; (**b**) negative input.

At positive half cycle as shown in Fig. 2a for i_s_ ≥ 0, q_d_ = 1

6 $$Vgo= Vdc/2$$

During the negative half-cycle, as seen in Fig. 2b for i_s_ < 0, q_d_ = 0

7 $$Vgo= -Vdc/2$$

where q_d_ = l, if Dl is on and q_d_ = 0, if D2 is on. The two capacitors must be adequately sized to function as a DC voltage source with little ripple, maintaining a nearly constant output.

**B. Inverter model:**

Table 1 delineates the eight operational states of the inverter switches. To maintain compliance with Kirchhoff’s current (KCL) and voltage (KVL) laws, it is imperative that both switches in the same branch are not activated simultaneously, since this would create a short circuit of the input voltage, contravening KVL. Thus, the two switches inside the same leg are complimentary.

The configuration specifies the inverter model as follows:

8 $$Sa1+Sa2=1$$

9 $$Sb1+Sb2=1$$

10 $$Sc1+Sc2=1$$

11 $$Vdc/2 (Sa1-Sa2) = VAn + Vno$$

12 $$Vdc/2 (Sb1-Sb2) = VBn + Vno$$

13 $$Vdc/2 (Sc1-Sc2) = VCn + Vno$$

where V_n0_ is the voltage that exists between the midpoint of the DC-link and the location (n) as shown in Fig. 1. Adding the Equations from (11) to (13) together gives Eq. (14) as

14 $$Vdc/2 (Sa1+Sb1+Sc1-Sa2-Sb2-Sc2) = VAn + VBn + VCn +3 Vno$$

As we are dealing with balanced voltages

15 $$VAn + VBn + VCn = 0$$

By substituting from Eqs. (8)–(13), (15) in Eq. (14)

16 $$Vdc/6 (2Sa1+ 2Sb1+2Sc1-3) = Vno$$

Substituting for V_no_ in Eqs. (11) to (13), gives phase voltages:

17 $$VAn = Vdc/3 (2Sa1- Sb1- Sc1)$$

18 $$VBn = Vdc/3 (2Sb1- Sa1- Sc1)$$

19 $$VCn = Vdc/3 (2Sc1- Sa1- Sb1)$$

Substituting for V_dc_ from Eqs. (6) and (7) in (17)–(19), gives phase voltages:

20 $$VAn = 2Vgo/3 (2Sa1- Sb1- Sc1)$$

21 $$VBn = 2Vgo /3 (2Sb1- Sa1- Sc1)$$

22 $$VCn = 2Vgo /3 (2Sc1- Sa1- Sb1)$$

**C. LC filter model:**

It is essential to construct an LC filter circuit within the three-phase VSI model to mitigate higher-order harmonics. LC filters must be engineered as low-pass filters to attenuate higher-order harmonics while remaining cost-effective, since the SPWM approach may mitigate harmonics of lower order. The inductor in the filter circuit must possess a current rating that is at least equal to or above the maximum value of the inverter output current. The three-phase currents at resistive load are shown in Eq. (23). Subscript x stands for phase a, b, or c.

$$\frac{\mathrm{d}}{\mathrm{d}\mathrm{t}}{\mathrm{i}}_{\mathrm{i},\mathrm{x}}\left(\mathrm{t}\right)= \frac{1}{{\mathrm{l}}_{\mathrm{f}}}[{\mathrm{v}}_{\mathrm{i},\mathrm{x}}\left(\mathrm{t}\right)- {\mathrm{v}}_{\mathrm{c}\mathrm{f},\mathrm{x}}\left(\mathrm{t}\right)]$$

$$\frac{\mathrm{d}}{\mathrm{d}\mathrm{t}}{\mathrm{v}}_{\mathrm{c}\mathrm{f},\mathrm{x}}\left(\mathrm{t}\right)= \frac{1}{{\mathrm{c}}_{\mathrm{f}}}*\frac{\mathrm{d}}{\mathrm{d}\mathrm{t}}\left[{\mathrm{i}}_{\mathrm{i},\mathrm{x}}\left(\mathrm{t}\right)- {\mathrm{i}}_{\mathrm{l},\mathrm{x}}\left(\mathrm{t}\right)\right]$$

23 $$\frac{\mathrm{d}}{\mathrm{d}\mathrm{t}}{\mathrm{i}}_{\mathrm{l},\mathrm{x}}\left(\mathrm{t}\right)= \frac{1}{{\mathrm{R}}_{\mathrm{l}}}[{\mathrm{v}}_{\mathrm{c}\mathrm{f},\mathrm{x}}\left(\mathrm{t}\right)- {\mathrm{v}}_{\mathrm{l},\mathrm{x}}\left(\mathrm{t}\right)]$$

where v_i_ is the output inverter voltage, i_i_ is the output inverter current, v_cf_ is filter capacitor voltage, i_l_ is load current, C_f_ is capacitor of filter, v_l_ is load voltage, and R_l_ is the load resistance.

## Modulation technique

**Fig. 3:**
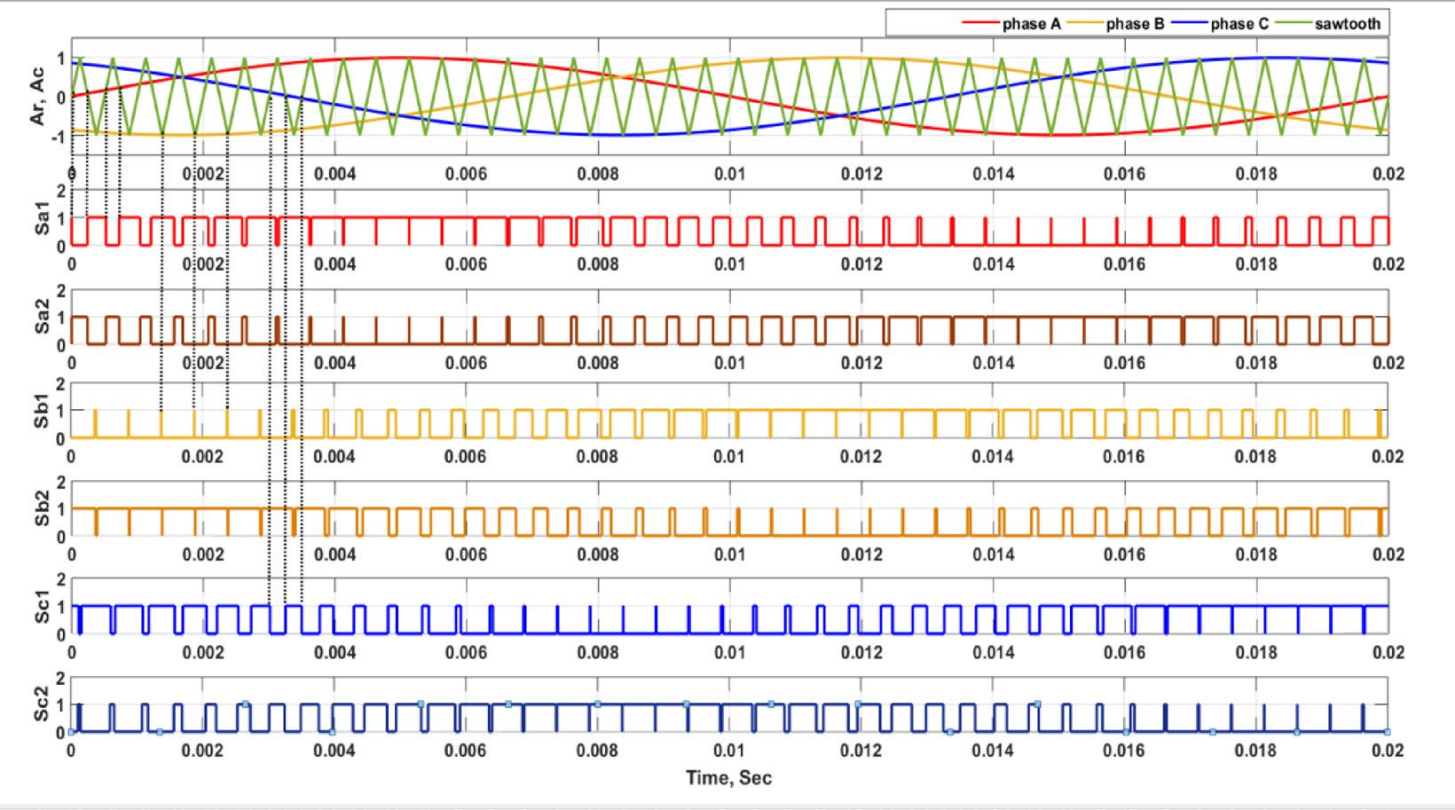
Modulation scheme.

**Fig. 4:**
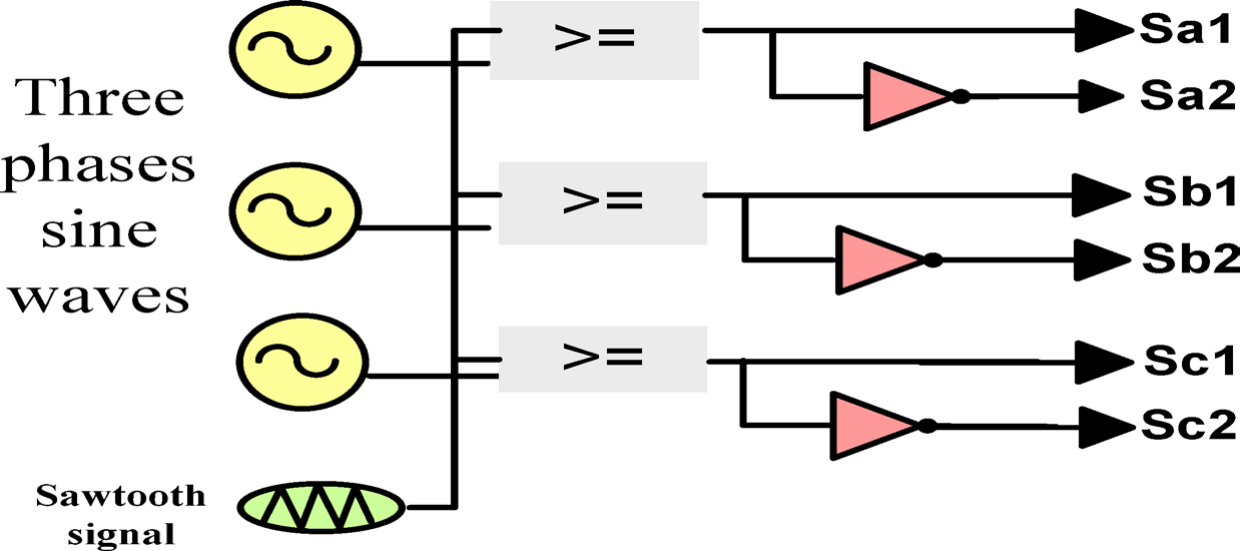
Gate signal logic pattern.

The pulse width modulation (PWM) approach is a preferred modulation scheme for power converters. Figure 3, depicts the sinusoidal PWM utilized in the suggested architecture and illustrates how the waveform of the gate signals is produced by the comparator and the toggling logic. SPWM is a prevalent technique in power electronic circuits for the digital control of switching commutation, owing to its simplicity^26,27^. The junction points of the carrier wave and references dictate the transition intervals. Logic circuits are used to produce six switching pulses by contrasting the sawtooth signal with three reference sine waves, as seen in Fig. 4. The three sine waves are needed as a reference signals and they are phase shifted by 120° with the desired output voltage frequency is taken and the switching devices will be ON whenever the reference sinusoidal signals become greater than the carrier triangular wave. According to Table 1, the result of this comparison is six pulses delivered to six switches via the logic diagram seen in Fig. 4. The modulation index (MI) of SPWM generator is the ratio of reference signal amplitude and carrier signal amplitude as given in eq.:

$${\mathrm{M}\mathrm{I} = \mathrm{A}}_{\mathrm{r}}/{A}_{C}$$

where, MI is modulation index of SPWM generator, A_r_ is reference signal amplitude, A_c_ is carrier signal amplitude. The modulation index of the SPWM generator needs to maintain in between 0 to 1. As high the value of MI, the THD will be less.

## Calculation of power losses and efficiency

In each semiconductor switch (IGBT or diode), there are three categories of power losses: conduction losses, switching losses, and blocking losses. When the switch is in the on-state, conduction losses are present, while switching losses are apparent during the switch’s on-and-off transition. With respect to the other two components, the blockage losses are negligible and may be disregarded^28^.

*(a) Conduction losses.*

The switch’s on-state resistance in the ON state is the cause of conduction losses. Equations (24) and (25) can be used to calculate the aforementioned losses for switches and diodes, respectively.

24 $${P}_{con\_sw}={V}_{sw\_on}*{i}_{s{w\_}_{avg}}+{R}_{s{w\_}_{on}}*{i}_{s{w\_}_{rms}}^{2}$$

25 $${P}_{con\_D}={V}_{D\_on}*{i}_{D\_avg}+{R}_{D\_on}*{i}_{D\_rms}^{2}$$

where P_con_sw_ indicates the conduction losses of the switch and P_con_D_ is the conduction losses of the diode. V_sw_on_ denotes switch voltage in the on state. V_D_on_ denotes the diode voltage in the on-state. The on-state resistance is ${R}_{s{w\_}_{on}}$ for the switch and R_D_on_ for the diode. i_sw_avg_, i_sw_rms_, i_D_avg_, and i_D_rms_ are the switch, diode average and RMS currents, respectively.

26 $$\mathrm{P}\mathrm{c}\mathrm{o}\mathrm{n}\mathrm{d}.(\mathrm{t}\mathrm{o}\mathrm{t}\mathrm{a}\mathrm{l})={P}_{con\_sw}+{P}_{con\_D}$$

*(b) Switching losses.*

Losses in switching are the consequence of non-instantaneous activation and deactivation processes. The current through the collector (I_C_) starts to rise when the voltage of the gate-emitter (V_GE_) surpasses the threshold voltage (V_T_) and the switch is activated. Furthermore, the collector-emitter voltage (V_CE_) begins to decline until it attains V_sw-on_, at which point an IC is achieved at I_sw-on_ within a t_on_. Equation (27)^23^, demonstrates that on-time switching losses occur during t_on_, whereas turn-off switching losses arise during t_off_.

The switch’s switching losses (P_sw_) can be articulated as:

$$\begin{aligned} P_{{SL,i\left( {on} \right)}} = & f_{cr} \mathop \smallint \limits_{0}^{{t_{on} }} V_{{sw\__{off} ,i\left( t \right)}} *i\left( t \right) dt \\ = & f_{cr} \mathop \smallint \limits_{0}^{{t_{on} }} \left( { - \frac{{V_{sw\_off,i} }}{{t_{on} }}\left( {t - t_{on} } \right)} \right)\left( {\frac{{I_{sw\_on,i} }}{{t_{on} }}t} \right)dt \\ = & {\raise0.7ex\hbox{$1$} \!\mathord{\left/ {\vphantom {1 6}}\right.\kern-0pt} \!\lower0.7ex\hbox{$6$}}f_{cr} *V_{sw\_off,i} *I_{sw\_on,i} *t_{on} \\ \end{aligned}$$

27 $$\begin{aligned} P_{{SL,i\left( {off} \right)}} = & f_{cr} \mathop \smallint \limits_{0}^{{t_{off} }} V_{{sw\__{off} ,i\left( t \right)}} *i\left( t \right) dt \\ = & f_{cr} \mathop \smallint \limits_{0}^{{t_{off} }} \left( {\frac{{V_{sw\_off,i} }}{{t_{off} }}t} \right)\left( { - \frac{{I_{sw\_off,i} }}{{t_{off} }}\left( {t - t_{off} } \right)} \right)dt \\ = & {\raise0.7ex\hbox{$1$} \!\mathord{\left/ {\vphantom {1 6}}\right.\kern-0pt} \!\lower0.7ex\hbox{$6$}}f_{cr} *V_{sw\_off,i} *I_{sw\_on,i} *t_{off} \\ \end{aligned}$$

where P_SL, i(ON)_ denotes the i_th_ switch’s turn-on switching loss and P_SL, i(OFF)_ denotes the i_th_ switch’s turn-off switching loss. The total switching losses are determined by summing the entire turn-on and turn-off losses for switches as per Eq. (28).

28 $${P}_{SL\left(total\right)}=\sum_{i=1}^{{N}_{sw}}\left(\sum_{j=1}^{{N}_{on(i)}}{P}_{SL,on(ij)}+\sum_{j=1}^{{N}_{off(i)}}{P}_{SL,off(ij)}\right)$$

where P_SL(Total)_ is characterized by the cumulative switching losses and N_sw_ is the total number of switches and N_s_on_ & N_s_off_ represent the counts of turn-ons and turn-offs during a single cycle in the suggested topology.

*(c) Converter efficiency.*

The power output of the converter can be represented as:

$${\mathrm{P}}_{{{\mathrm{o}}({\mathrm{phase}})}} = {\text{ R}}_{{\mathrm{o}}} *{\text{ I}}^{{2}}_{{{\mathrm{o}} - {\mathrm{rms}}}}$$

29 $${\mathrm{P}}_{{\mathrm{o}}} = {3}*{\text{ P}}_{{{\mathrm{o}}({\mathrm{phase}})}}$$

The losses power of the converter represents the aggregate of losses in the switches and losses associated with passive components, as defined in Eqs. (26) and (28).

30 $$\mathrm{P}\mathrm{C}\mathrm{o}\mathrm{n}\mathrm{v}\mathrm{e}\mathrm{r}\mathrm{t}\mathrm{e}\mathrm{r} \mathrm{l}\mathrm{o}\mathrm{s}\mathrm{s}\mathrm{e}\mathrm{s} = \mathrm{P}\mathrm{c}\mathrm{o}\mathrm{n}\mathrm{d}. (\mathrm{t}\mathrm{o}\mathrm{t}\mathrm{a}\mathrm{l}) + {P}_{SL\left(total\right)}$$

The input power of the converter can be represented as:

31 $${\mathrm{P}}_{{{\mathrm{in}}}} = {\text{ P}}_{{\mathrm{o}}} + {\text{ P}}_{{\text{Converter losses}}}$$

The converter’s efficiency % can be determined using Eqs. (29) and (31) as follows:

32 $${\mathrm{Efficiency}}\% = {\mathrm{P}}_{{\mathrm{o}}} /{\mathrm{P}}_{{{\mathrm{in}}}} \times 100$$

## Comparison study of single phase to three phase converter

A comparison study is carried out on the proposed converter configuration with different single-phase to three-phase configurations to focus on the advantages of the proposed topology. The parameters of comparison are done for component counts (switches, diodes, capacitors, inductors), voltage ripple of DC link capacitors, the THD of input current, gain and modulation control technique as depicted in Table 2. The proposed circuit necessitates six switches, which is less than the topologies in^10,13,14,17,24,25^. The all topologies which are compared with the proposed topology do not provide an output voltage gain despite using a greater number of switches and passive components. The input current THD is a low value compared to topologies^13–15^. Conduction losses were reduced as the switch count was reduced. DC-link voltage ripple is a low value compared to topologies of^17,24,25^. The compared topologies have boosting factor of the DC link voltage and a higher count of components which lead to high cost of the converters^13–15,17^ due to high TSV which illustrated by V_dc_/V_s_. The modulation technique is simple approach rather than Space Vector Pulse Width Modulation (SVPWM) which used in the compared topologies.

**Table 2:** Performance analysis of different topologies.

N_leg_	^10^ 2016	^13^ 2021	^14^ 2023	^15^ 2018	^17^ 2021			^24^ 2018	^25^ 2021	pc
	10L	7L	7L	2L	4L	5L	P5L	5L	5L	3L
Nsw	20	14	14	4	8	10	10	10	10	6
N_D_	0	0	0	4	0	0	0	0	0	2
N_C_	4	2	2	2	2	2	2	1	1	2
N_L_	4	4	4	1	2	1	2	4	4	1
DC-link voltage ripple (V_max_ − V_min_)/V_dc_	0.1	2.1	2.1	3	8.1	6.25	6.97	13.3	13.3	3.3
Input current THD	–	6.94%	6.94%	30.34%	5.56%	0.77%	4.05%	2.3%	2.3%	6.85%
Output voltage gain	Not count	Not count	Not count	Not count	Not count	Not count	Not count	Not count	Not count	Boosting 2
V_dc_/V_s_	1	1.29	1.29	1.5	1.189	1.028	1.446	Not count	Not count	0.75
Modulation control technique	Undefined	SVPWM	SVPWM	Undefined	SVPWM			Undefined	Undefined	SPWM

N_sw_, number of switches; N_D_, number of diodes (excluding those in antiparallel and series configurations with an IGBT); N_c_, capacitors numbers; N_L_, number of inductors; V_s_, supply voltage; [pc], proposed converter.

Therefore, the major purpose of this research is to minimize the number of switches and component count and thus minimize the cost of the suggested converter with a comparison of different topologies. It reduces the voltage ripple of DC link capacitors while maintaining low harmonics in the input and output currents. Thus, the lower value of voltage stresses of all switches leads to decrease the value of converter cost. Moreover, the input power factor can be improved due to decrease THD, which can be ascertained from the THD of the input current as:

$$\mathrm{P}\mathrm{F} =\frac{1}{\sqrt{1+ {\left(\frac{{\%THD}_{i}}{100}\right)}^{2}}}cos\varphi$$, and %THD_i_ = $$\frac{\sqrt{{I}_{2}^{2}+{I}_{3}^{2}+{I}_{4}^{2}+{I}_{5}^{2}+\dots .+{I}_{n}^{2}}}{{I}_{1}}$$,

where, I_1_ is RMS value of the fundamental current, I_2_, I_3_, …, I_n_ are RMS value of 2^nd^, 3^rd^, through n^th^ harmonic currents.

## Simulation results

The proposed converter model is simulated using the MATLAB/Simulink program. The converter parameters are specified in Table 3, and the circuit’s efficacy is assessed at a switching frequency of fsw = 2 kHz.

**Table 3:** Parameters of the proposed converter.

Circuit parameter	Simulation and Experimental
Input voltage	80, 60 V/ 50 Hz
Source impedance	1 Ω, 50mH
DC-link capacitors C1 = C2	4700 μF
Output filter inductance	25 mH
Output filter capacitance	330 μF
Resistive load	550 Ω, 1 kΩ
Switching frequency(f_sw_)	2000 Hz

**Fig. 5:**
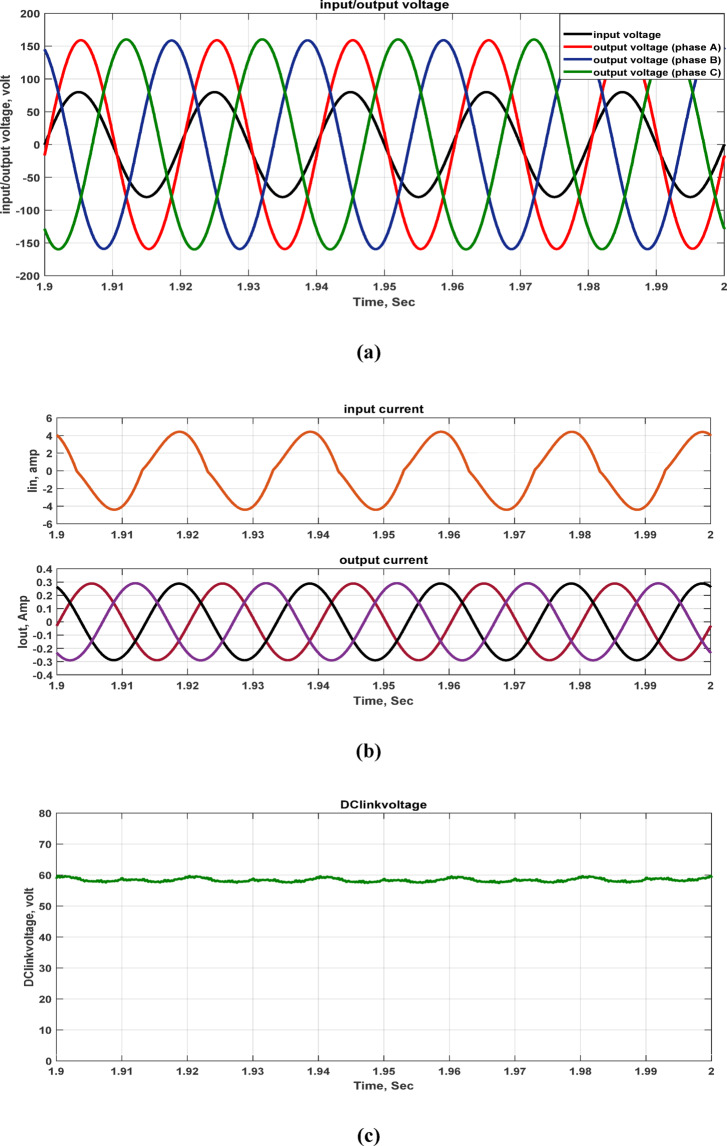
Simulation outcomes of the suggested converter at R-load = 550 Ω and f_sw_ = 2 kHz supplying a resistive load: (**a**) Input single-phase voltage and output three-phase voltage, (**b**) input single-phase current and output three-phase current, and **c** DC-link voltage.

The system operates on an 80 V AC supply and is linked to a resistive load. Figure 5 illustrates the input and output voltages, and input and output currents, and DC-link voltage, with the resistive load set to 550 Ω. At an input voltage of 80 V, the output voltage per phase is 160 V, with a voltage gain that is twice the input supply. This suggests that the voltage gain is approximately twice as high as the input voltage.

**Fig. 6:**
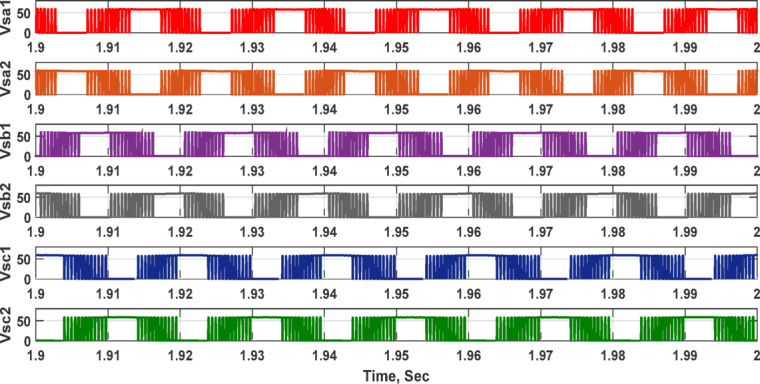
Simulation outcomes of the suggested converter at R-load = 550 Ω and f_sw_ = 2 kHz supplying a resistive load: voltage stresses of all switches Vsa1, Vsa2, Vsb1, Vsb2, Vsc1, and Vsc2.

Furthermore, the output voltage and current waveforms are three-phase with nearly balanced and sinusoidal waveforms. In Fig. 5c shows the DC link voltage which is nearly DC that depends on DC link capacitor values. The voltage stresses across all switches at the same load are illustrated in Fig. 6. The greatest switch voltage nearly equals 60 V which is less than the input voltage.

**Fig. 7:**
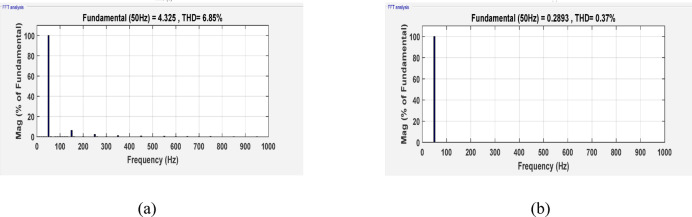
Simulation THD spectrum (**a**) input current and (**b**) output current, f_sw_ = 2000Hz.

**Fig. 8:**
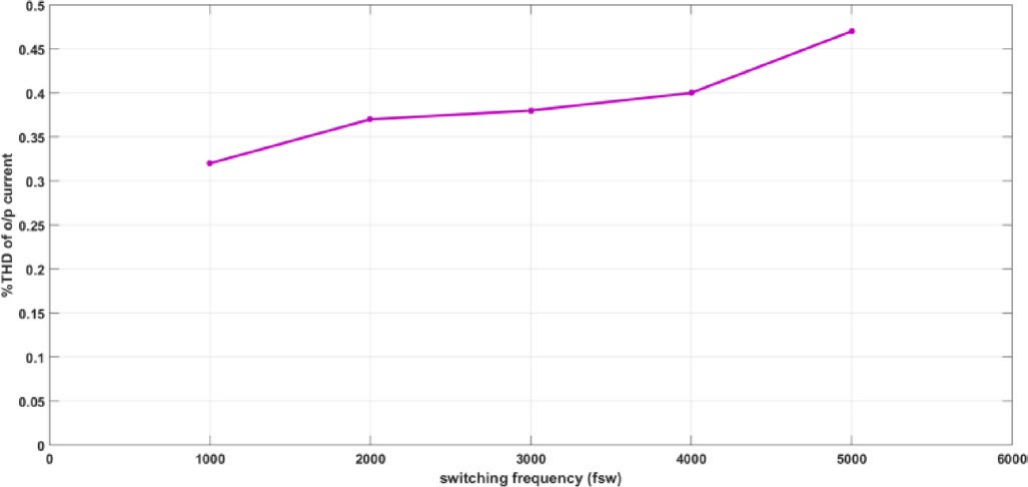
Simulation output current THD of the proposed converter for various switching frequencies.

Figure 7a and b illustrate the FFT analysis of the input and output currents, respectively that show the effect of filter parameters. THD of input current and output current are 6.85% for input current and 0.37% for output current that with IEEE standard equals 8%. The standard IEEE is referred in IEEE standard for harmonic control in electric power systems (IEEE Std 519™-2022). THD of the output current with various switching frequencies are demonstrated in Fig. 8 which illustrates that the waveforms are nearly sinusoidal with low THD in all switching frequencies.

**Fig. 9:**
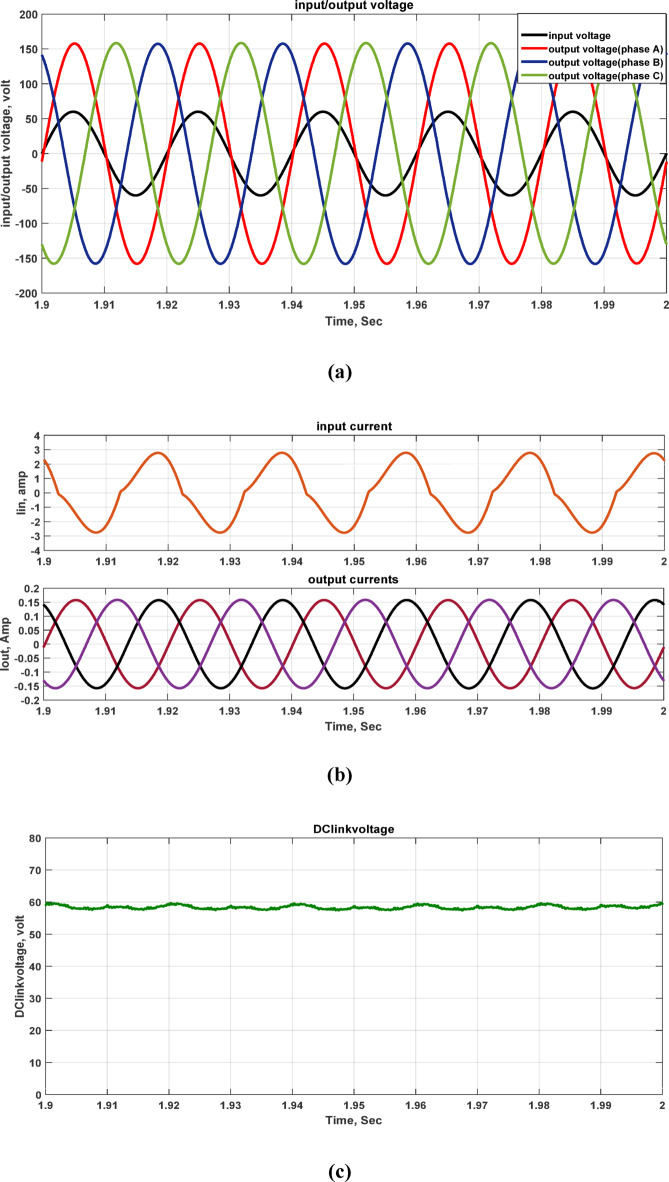
Simulation outcomes of the suggested converter at R-load = 1000 Ω, and f_sw_ = 2 kHz supplying a resistive load: (**a**) Input single-phase voltage and output three-phase voltage, (**b**) input single-phase current and output three-phase current, and (**c**) DC-link voltage.

A case study is evaluated for load and input voltage variation which are (1000 Ω, 60V) at (f_sw_) = 2000 Hz. Figure 9 illustrates the input voltage, output voltage, input current, output current, and DC-link voltage. When the load is changed to 1000 Ω and the input voltage is decreased to 60V, the output voltage still equals 160 V. Thus, the inverter’s voltage gain is increased from 2 to 2.71 gain at the same other parameters. The voltage stresses across all switches at the same load are illustrated the same value as the previous case in Fig. 6. The greatest switches voltage nearly equals 60 V which is similar to the DC link voltage.

Thus, the three-phase output voltages and currents waveforms are sinusoidal and almost balanced. Moreover, the values of the DC link capacitors determine the DC link voltage, which is approximately DC with low value of ripples.

## Experimental results

**Fig. 10:**
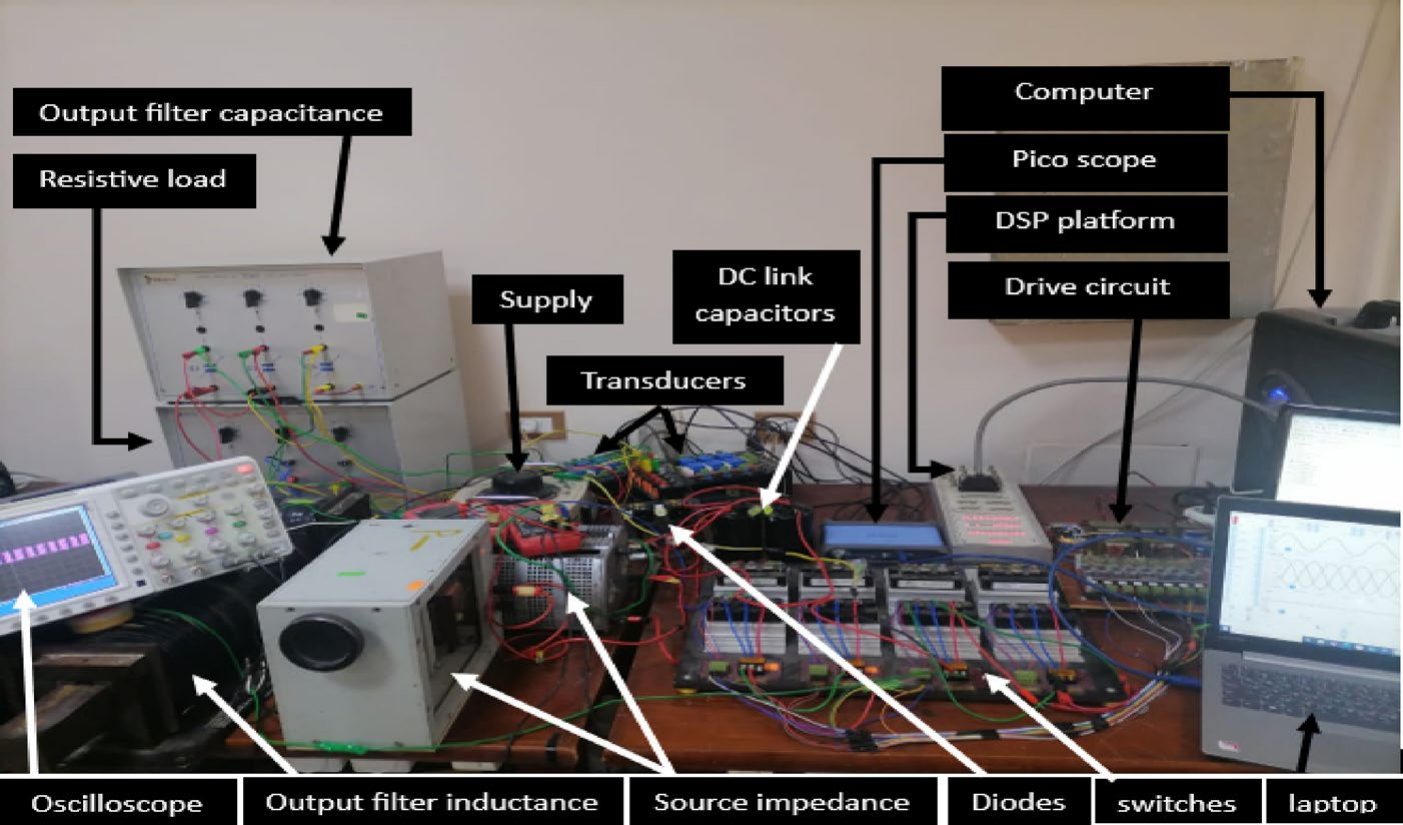
Experimental setup of the overall system.

The experimental configuration makes use of six IGBT switching devices. The design of the inverter utilized IGBT modules of the type (MITSUBISHI CM100DY-24H). (dSPACE-1104) is used to implement the control system. Analog-to-digital converters relay the output current measurements from the LA25-NP sensors to the dSPACE-1104 platform. Additionally, sensors are used to measure the output voltage and capacitor voltage. The drive pulse power obtained from (dSPACE—1104) is amplified via an interface circuit to a higher level sufficient for controlling the inverter’s gates and separating the control system from the power circuit. Furthermore, the Picoscope 4000 series uses voltage and current transducers to record the results. The experimental setup for the whole system is illustrated in Fig. 10.

**Fig. 11:**
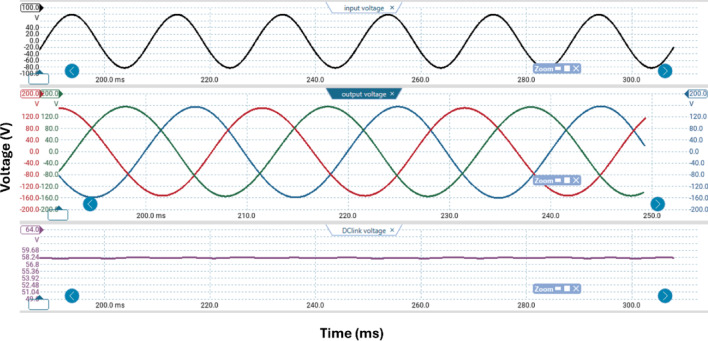
Experimental outcomes of the suggested converter at R-load = 550 Ω, and f_sw_ = 2 kHz supplying a resistive load including input single-phase voltage and output three-phase voltage, and DC-link voltage.

**Fig. 12:**
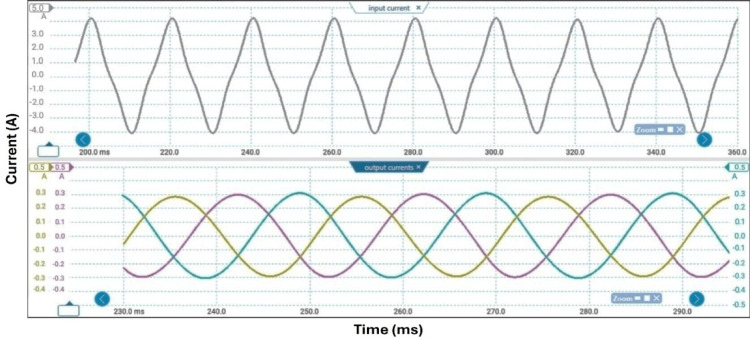
Experimental outcomes of the suggested converter at R-load = 550 Ω, and f_sw_ = 2 kHz supplying a resistive load are as follows: currents of input single-phase and output three-phase.

**Fig. 13:**
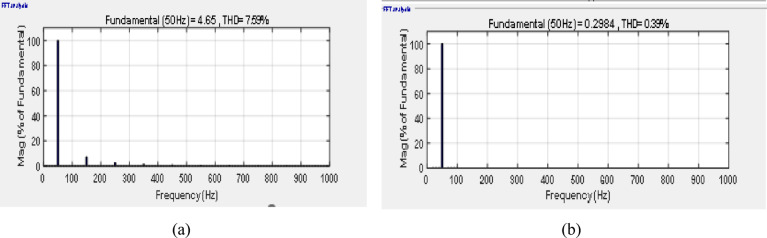
Experimental THD spectrum (**a**) input current and (**b**) output current, f_sw_ = 2000Hz.

**Fig. 14:**
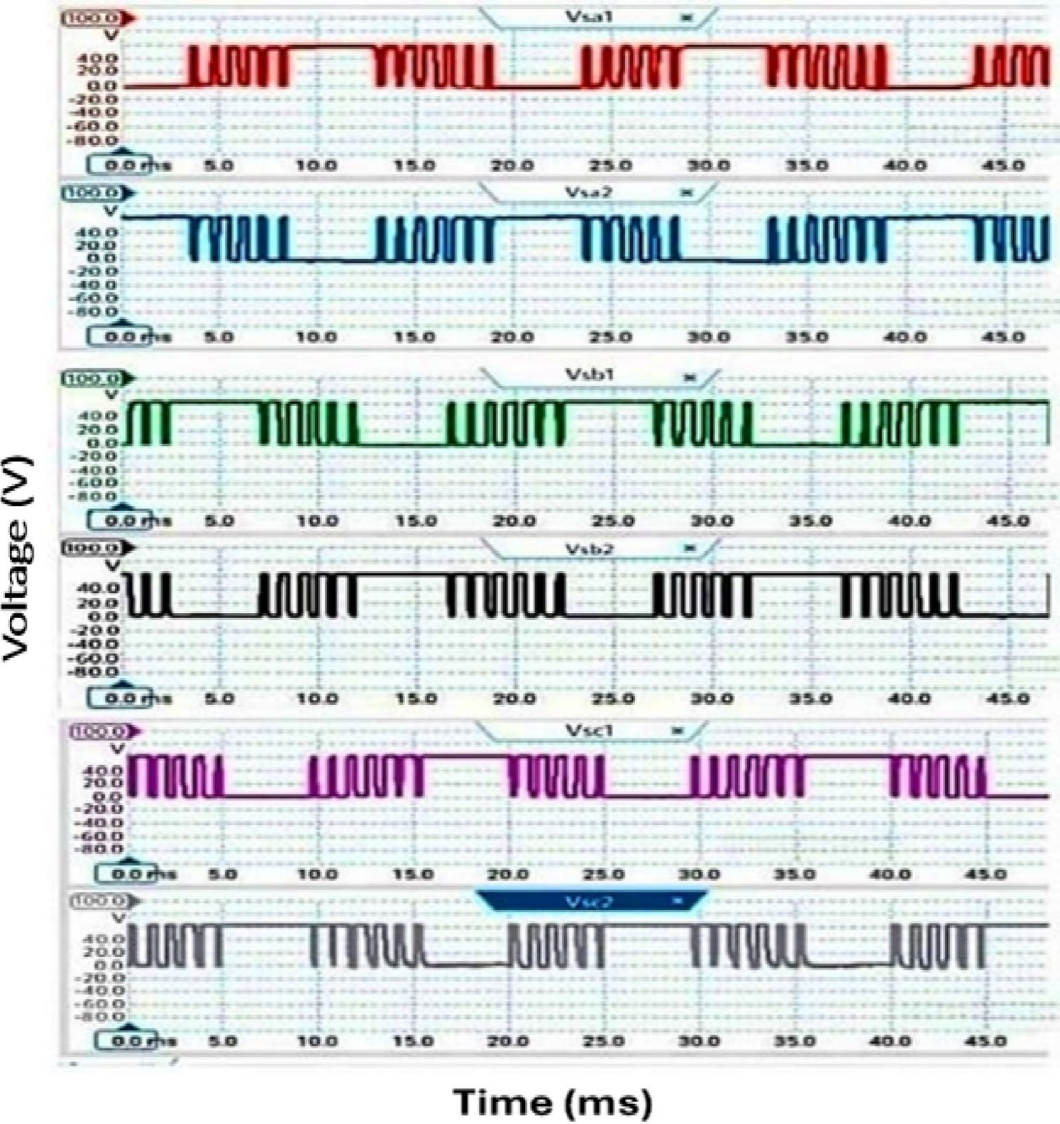
Voltage across switches, f_sw_ = 2000Hz.

**Fig. 15:**
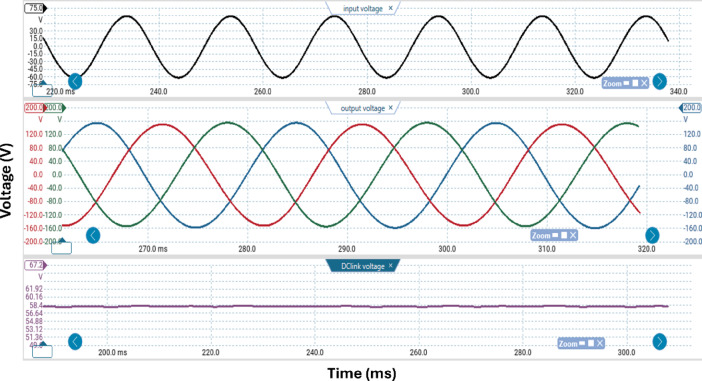
Experimental outcomes of the suggested converter at R-load = 1000 Ω, and f_sw_ = 2 kHz supplying a resistive load including input single-phase voltage and output three-phase voltage, and DC-link voltage.

**Fig. 16:**
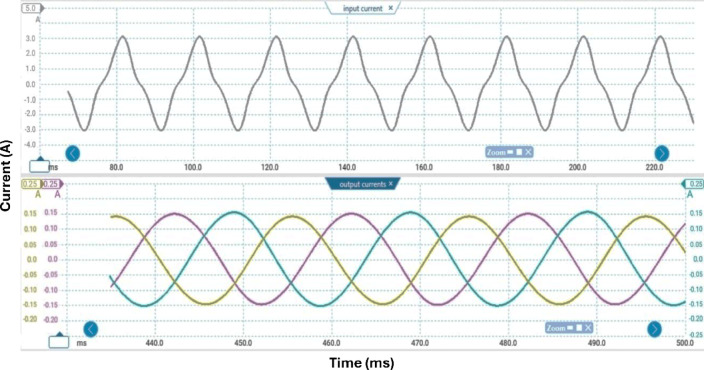
Experimental outcomes of the suggested converter at R-load = 1000 Ω, and f_sw_ = 2 kHz supplying a resistive load are as follows: currents of input single-phase and output three-phase.

**Fig. 17:**
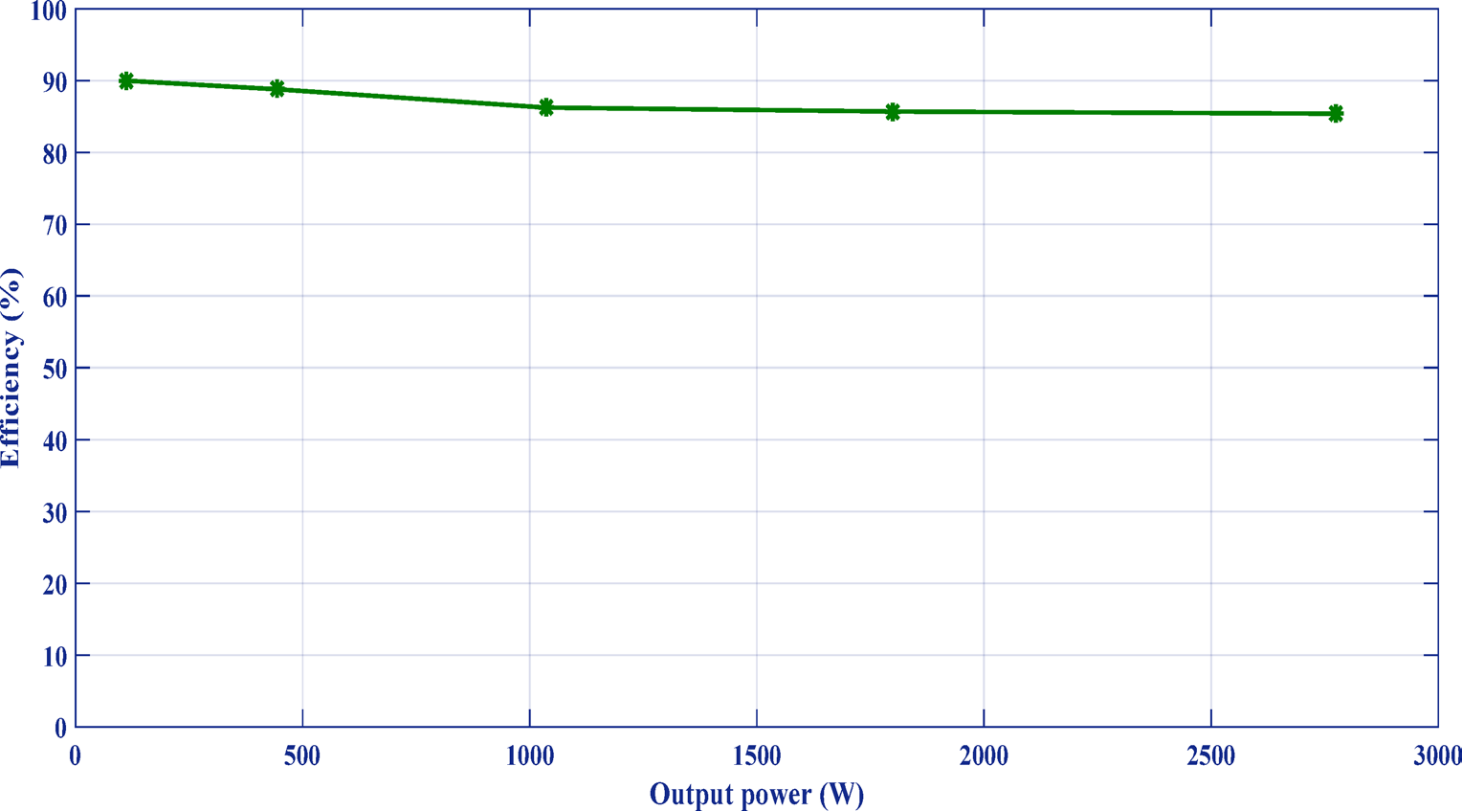
Efficiency of the proposed converter for various load power levels.

Table 3 lists the parameters that were employed to validate the suggested topology prototype, which are identical to those in the simulation case. The Pico scope demonstrates the peak value of input AC voltage is 80 V in Fig. 11, and the corresponding load voltages of three phases, and DC link voltage at R-load are shown at (f_sw_) 2000 Hz. The DC link voltage is self-balanced at V_dC_ = 60 V. The output voltage is 160 Volt which confirms twice the voltage gain. Figure 12 demonstrates the results for the currents of input and output at the same load and switching frequency. It is detected that THD of the currents of input and output for (f_sw_) 2000 Hz within acceptable limits as shown in Fig. 13. Figure 14 depicts the voltage across all switches of the three phases according to the input voltage of 80 Volt. The voltage stress of each switch equivalents to the DC link voltage which is 60 V. Figures 15 and 16 illustrate the similar results for another input voltage 60 V and resistance load equals 1000 Ω at the same frequency of switching. The output voltage is the same voltage of the previous case which confirms 2.7 the voltage gain. From Figs. 11, 12, 13, 14, 15 and 16, it is noticed that the supply current is nearly sinusoidal, and the load voltages are three phase balance voltage which is boosted for two time and more than the input voltage. The DC link voltage is nearly DC with low ripple value. The suggested converter has an efficiency of 90% when the input voltage is 80 V, the switching frequency is 2 kHz, and the load resistance is 500 Ω. As shown in Fig. 17, the efficiency of the recommended converter varies with the different output power. The high efficiency of the suggested converter can be attributed to the utilization of fewer passive components and semiconductor switches. By decreasing the power electronics components, the converter’s overall size, power losses, and cost can all be decreased.

## Conclusion

This work introduces a boost AC–AC SPTTP converter that utilizes a smaller number of semiconductor switches and a minimal quantity of passive components. Consequently, the converter’s efficacy increases and ranged between 85 to 90% according to the output power which is reached to 2700W, and its size and power losses decrease. The circuit analysis is executed with accurate details. The converter that has been recommended is preferable to other converters, as evidenced by a comparison analysis with previous models. The system is operated on two cases in the simulation and the experimental. At an input voltage of 80 V, the output voltage per phase is 160 V, with a voltage gain that is twice the input supply. Moreover, at another case, the input voltage of 60 V and resistance load equals 1000 Ω at the same frequency of switching. The output voltage is the same voltage of the previous case which confirms 2.7 the voltage gain. The switch’s voltage stresses are 60V which equal to the DC link voltage. The input and output waveforms exhibit adequate levels of total harmonic distortion. The system’s performance is shown by simulation studies, which show that the converter’s input and output currents are approximately sinusoidal with total harmonic distortion (THD) 6.85% and 0.37%, respectively, under variable load conditions. The proposed topology has been verified in a variety of contexts by both the experimental apparatus and the simulation evaluation. A remarkable level of concordance has been demonstrated by the modeling and experimental results of the proposed circuit, which has been effectively constructed.

## Supplementary Information

Below is the link to the electronic supplementary material.

Supplementary Material 1

## Data Availability

The datasets used and/or analyzed during the current study available from the corresponding author on reasonable request.
